# Early neonatal mortality in twin pregnancy: Findings from 60 low- and middle-income countries

**DOI:** 10.7189/jogh.08.010404

**Published:** 2018-06

**Authors:** Saverio Bellizzi, Howard Sobel, Ana Pilar Betran, Marleen Temmerman

**Affiliations:** 1 World Health Organization, Western Pacific Regional Office, Manila, Philippines; 2 UNDP/UNFPA/UNICEF/WHO/World Bank Special Programme of Research, Development and Research Training in Human Reproduction, Department of Reproductive Health and Research, World Health Organization, Geneva, Switzerland; 3 Department of Obstetrics and Gynaecology, Ghent University, Ghent, Belgium; 4 Aga Khan University East Africa, Nairobi, Kenya

## Abstract

**Background:**

Around the world, the incidence of multiple pregnancies reaches its peak in the Central African countries and often represents an increased risk of death for women and children because of higher rates of obstetrical complications and poor management skills in those countries. We sought to assess the association between twins and early neonatal mortality compared with singleton pregnancies. We also assessed the role of skilled birth attendant and mode of delivery on early neonatal mortality in twin pregnancies.

**Methods:**

We conducted a secondary analysis of individual level data from 60 nationally-representative Demographic and Health Surveys including 521 867 singleton and 14 312 twin births. We investigated the occurrence of deaths within the first week of life in twins compared to singletons and the effect of place and attendance at birth; also, the role of caesarean sections against vaginal births was examined, globally and after countries stratification per caesarean sections rates. A multi-level logistic regression was used accounting for homogeneity within country, and homogeneity within twin pairs.

**Results:**

Early neonatal mortality among twins was significantly higher when compared to singleton neonates (adjusted odds ratio (aOR) 7.6; 95% confidence interval (CI) = 7.0-8.3) in these 60 countries. Early neonatal mortality was also higher among twins than singletons when adjusting for birth weight in a subgroup analysis of those countries with data on birth weight (n = 20; less than 20% of missing values) (aOR = 2.8; 95% CI = 2.2-3.5). For countries with high rates (>15%) of caesarean sections (CS), twins delivered vaginally in health facility had a statistically significant (aOR = 4.8; 95% CI = 2.4-9.4) increased risk of early neonatal mortality compared to twins delivered through caesarean sections. Home twin births without SBA was associated with increased mortality compared with delivering at home with SBA (aOR = 1.3; 95% CI = 1.0-1.8) and with vaginal birth in health facility (aOR = 1.7; 95% CI = 1.4-2.0).

**Conclusions:**

Institutional deliveries and increased access of caesarian sections may be considered for twin pregnancies in low- and middle- income countries to decrease early adverse neonatal outcomes.

The amount and quality of research on twins varies greatly worldwide, with the majority of epidemiological evidence coming from high-income countries [1,2]. Reliable information of incidence of twins is available for most high-income countries through birth registration, and ranges from 14.6 per thousand live births in South Korea up to 21.2 per thousand live births in Denmark [3]; however, low and middle-income countries (LMIC) are less apt to have accurate statistics [4]. Rates of twinning vary from 6-9 per thousand live births in South and South-East Asian Region and to above 20 per thousand live births in Central African countries [2,5-8].

Multiple pregnancies in low-resource settings pose women and newborns at increased risk of death due to poorer management of multiple pregnancies and management of post-partum haemorrhage, preeclampsia and preterm birth which occur more commonly with multiple pregnancies [9-11]. The relation between survival of mothers and their twinned newborns and mode of delivery (vaginal vs caesarean section) is not clear, particularly in these settings [12,13].

We analysed individual level data from 60 countries using the Demographic and Health Surveys (DHS) Program. We included data from low- and middle-income countries with the aim to assess early neonatal mortality in twins compared with singleton neonates, as well as the role of place and assistance at birth, and route of delivery.

## METHODS

### Study design

We conducted an analysis based on publicly available data sets from the Demographic and Health Surveys (DHS) [14]. We included data from 60 countries which represented the latest country DHS over the last 15 years with available data on mortality and caesarean sections in single and multiple pregnancies (**Table 1**).

**Table 1:** List of countries and years under study

African Region	American Region	Eastern Mediterranean Region	European Region	South East Asian Region	Western Pacific Region
Benin 2011/12	Bolivia 2008	Egypt 2014	Albania 2008/09	Bangladesh 2011	Cambodia 2010
Burkina Faso 2010	Colombia 2010	Jordan 2012	Armenia 2010	India 2005/06	Philippines 2013
Burundi 2010	Dominican Republic 2013	Morocco 2003/04	Azerbaijan 2006	Indonesia 2012	
Cameroon 2011	Guyana 2009	Pakistan 2012/13	Kyrgyz Republic 2012	Maldives 2009	
Chad 2004	Haiti 2012		Moldova 2005	Nepal 2011	
Comoros 2012	Honduras 2011/12		Taijikistan 2012	Timor Leste 2009/10	
Congo (Brazzaville) 2011/12	Peru 2012		Turkey 2003		
Congo (Democratic Republic) 2013/14			Ukraine 2007		
Cote d Ivoire 2011/12					
Ethiopia 2011					
Gabon 2012					
Gambia 2013					
Ghana 2008					
Guinea 2012					
Kenya 2008/09					
Lesotho 2009					
Liberia 2013					
Madagascar 2008/09					
Malawi 2010					
Mali 2012/13					
Mozambique 2011					
Niger 2012					
Nigeria 2013					
Rwanda 2010					
Sao Tome and Principe 2008/09					
Senegal 2014					
Sierra Leone 2013					
Swaziland 2006/07					
Tanzania 2010					
Togo 2013/14					
Uganda 2011					
Zambia 2007					
Zimbabwe 2010/11					

DHS are cross-sectional nationally-representative household surveys that provide data for a wide range of maternal and infant health and nutrition indicators [14]. With more than 300 surveys in 90 countries, the DHS program is considered the best available way of obtaining cross-sectional information on health indicators in developing countries. In these surveys, women are interviewed about their reproductive history with survival of their offspring as well as their personal and household socioeconomic characteristics. Standard DHS surveys have large sample sizes (usually between 5 000 and 30 000 households) and typically are conducted about every 5 years, to allow comparisons over time. They are conducted by trained personnel using a standardized questionnaire and strict methods for sampling and data collection. The figures obtained from the DHS refers to births that occurred up to 5 years previous to the data of the survey [14].

### Study population

We included all singleton and twin births over the five years preceding from the most recent standard country-survey within the last fifteen years. We excluded triplets and higher order multiple births as well as all neonatal deaths occurring after the first week of life. We merged country data sets into one cross-sectional database.

### Outcome and exposures

Our main outcome was death during the first week of life (days 0-6, “early neonatal deaths”).

We explored the association between the main outcome and the type of pregnancy regarding the number of foetuses (singleton vs twin pregnancies). Additionally, we examined the association between mode of delivery (caesarean section vs vaginal delivery) and early neonatal mortality separately for singleton and twins for those births taking place in health facilities. Subgroup analysis was performed after stratifying countries according to overall caesarean section rates as low (<5%), medium (5%-15%), and high (>15%) [15].

We also investigated the result of place and attendance at birth on early mortality for the twins under study. Place and attendance at birth were based on women reports and were categorized as follows: births at home without skilled birth attendance (SBA), births at home with SBA and births in health facilities.

In addition, the association between early neonatal mortality in twins and low birth weight (LBW) was studied, with LBW defined as weight at birth less than 2500 g.

In the logistic regression, we adjusted for the following co-variates: a wealth index [16] derived from an index of household assets, the number of antenatal visits, mother’s education, maternal age at birth of child, parity and previous birth interval categorized in “less than 18 months”, “18-23 months”, “18-35 months” and “more than 35 months”.

### Statistical analysis

**Figure 1:**
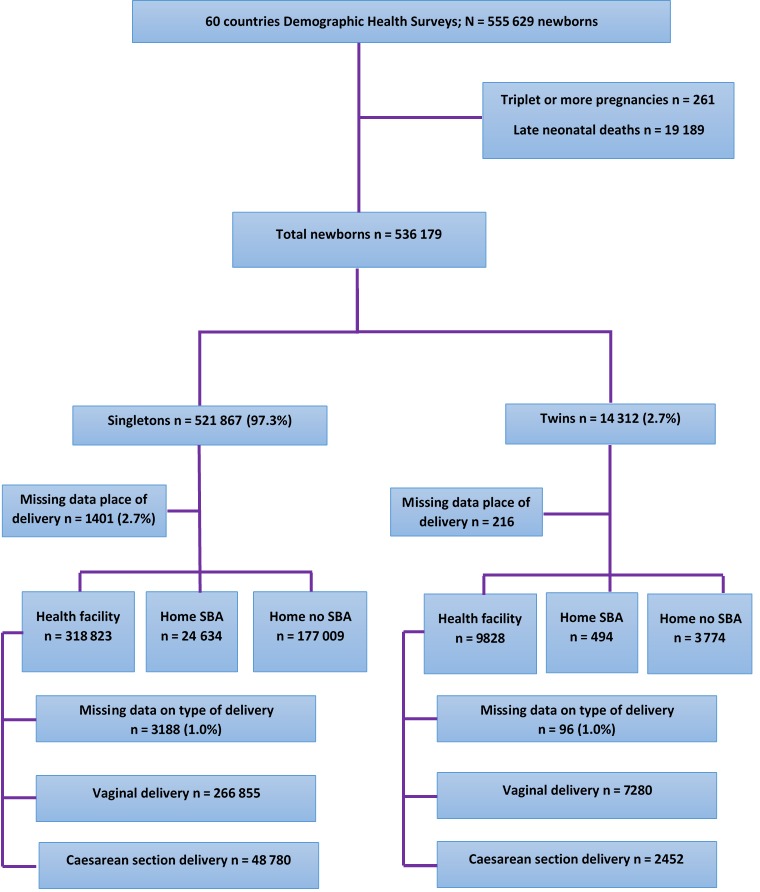
Study participants flowchart.

After the exclusion of triplets and higher order of multiple pregnancies and the exclusion of late neonatal deaths, 536 179 births, 521 867 singletons and 14 312 twins, were eligible for this analysis (**Figure 1**).

We initially tabulated the distribution of livebirths and early neonatal deaths, singletons and twin births by country and by World Health Organization (WHO) region. WHO classifies the 194 Member States in six regions: African Region (AFR, n = 47), Region of the Americas (AMR, n = 35), South-East Asia Region (SEAR, n = 11), European Region (EUR, n = 53), Eastern Mediterranean Region (EMR, n = 21), and Western Pacific Region (WPR, n = 27). We also tabulated the distribution of caesarean sections as well as place and attendance at delivery for all singleton and twin pregnancies by country, WHO region and the total.

We conducted a descriptive analysis of selected maternal and delivery characteristics in singleton and twin pregnancies.

A logistic regression was performed to calculate unadjusted and adjusted odds ratio for the association between early neonatal mortality and type of pregnancy (singleton and twin). The pooled OR for early neonatal mortality among twins vs singletons was adjusted for the following confounders: the presence of at least one antenatal care visit, mode of delivery (vaginal/caesarean section), household wealth (as a proxy for socio-economic status), and other birth-related confounding variables like birth spacing [17,18].

The pooled OR for early newborn mortality was adjusted for these confounders. We used a random effect model to control for unobserved factors at primary sampling unit and country levels [19].

The model also accounted for the clustering of twins within mothers, which is often overlooked and can affect the precision of estimates [20].

In consideration of the likely confounding effect of birthweight in the association of early neonatal death with singleton/twin pregnancies we conducted a subgroup analysis using logistic regression on the pooled data set of 20 countries having less than 20% of missing data on weight at birth. These are Albania 2008/09, Armenia 2010, Azerbaijan 2006, Bolivia 2008, Congo (Brazzaville) 2011/12, Dominican Republic 2013, Gabon 2012, Guyana 2009, Honduras 2011/12, Indonesia 2012, Jordan 2012, Kyrgyz Republic 2012, Maldives 2009, Moldova 2005, Peru 2012, Philippines 2013, Sao Tome and Principe 2008/09, Swaziland 2006/07, Taijikistan 2012, Ukraine 2007.

Data on the type of birth (vaginal or CS) for health facility births was available for 9 732 twins (99.0%) and for 315 635 singletons (99.0%).

To explore the association between early neonatal mortality and place and attendance at birth for the twin pregnancies population, 14 096 newborns (98.5%) with data on the exposure variables remained.

Throughout the analysis, p-values of <0.05 were considered significant. Statistical analysis was performed with STATA 13.1 SE (StataCorp LP, College Station TX, USA) [21].

### Ethical approval

This study used existing data obtained from ORC Macro through formal request mechanisms. No additional ethical review for the secondary analysis was required since each country and the institutional review board of ORC Macro (Calverton, MD, USA) approved the DHS data collection procedures.

## RESULTS

Sixty countries were included in this analysis; 33 countries from AFR, 8 from EUR, 7 from AMR, 6 from SEAR, 4 from EMR and 2 from WPR (**Table 1**). The study population included 536 179 livebirths (521 867 singletons and 14 312 twins) and ranged from 1 208 newborns in the 2007 Ukraine DHS to 50 026 in the 2005/06 India DHS. Africa contributed the largest number of newborns (56% of the total sample size in this analysis) followed by SEAR with 18%.

**Table 2** shows by country the number of livebirths (singletons and twins) contributing to the analysis, and the early neonatal mortality in each group of newborns with regional averages. The lowest country percentages of twin-pairs were registered in the Bolivia, Moldova and Nepal surveys with rates of 6 per thousand births; the highest rates were found in Africa: Benin, Cameroon, Comoros, Congo (Brazzaville), Cote d’Ivoire, Ghana, Sao Tome and Principe and Togo, which reported 20 or more twin-pairs per thousand births (**Table 2**). By WHO region, highest rates of twin pregnancies were found in AFR (1.65%) followed by EMR and EUR with 0.9% and 0.85%, respectively.

**Table 2:** Number of total newborns, singleton, twin, early neonatal deaths in singletons and in twins in the five years preceding 60 low and middle-income Demographic and Health Survey (DHS) countries between 2000 and 2014, by WHO Region

Country, survey years	N live births	Singletons, N (%)	Twins, N (%)	Singletons early neonatal deaths (%)	Twins early neonatal deaths (%)
**European Region (EUR)**	**21 715**	**21 349 (98.3)**	**366 (1.7)**	**269 (1.3)**	**34 (9.3)**
Albania 2008/09	1 598	1 562 (97.8)	36 (2.2)	12 (0.8)	3 (8.3)
Armenia 2010	1 458	1 438 (98.6)	20 (1.4)	8 (0.6)	0 (0.0)
Azerbaijan 2006	2 247	2 219 (98.8)	28 (1.2)	54 (2.4)	2 (7.1)
Kyrgyz Republic 2012	4 314	4 234 (98.1)	80 (1.9)	59 (1.4)	8 (10.0)
Moldova 2005	1 539	1 521 (98.8)	18 (1.2)	6 (0.4)	0 (/)
Tajikistan 2012	4 919	4 823 (98.0)	86 (2.0)	60 (1.2)	12 (14.0)
Turkey 2003	4 442	4 370 (98.4)	72 (1.6)	63 (1.4)	8 (11.1)
Ukraine 2007	1 208	1 182 (97.8)	26 (2.2)	7 (0.6)	1 (3.8)
**South East Asian Region (SEAR)**	**94 736**	**93 348 (98.5)**	**1388 (1.5)**	**2065 (2.2)**	**203 (14.6)**
Bangladesh 2011	8 575	8 431 (98.3)	144 (1.7)	209 (2.5)	24 (16.7)
India 2005/06	50 026	49 328 (98.6)	698 (1.4)	1267 (2.6)	101 (14.4)
Indonesia 2012	17 671	17 407 (98.5)	264 (1.5)	268 (1.5)	40 (15.2)
Maldives 2009	3 793	3 737 (98.5)	56 (1.5)	26 (0.7)	7 (12.5)
Nepal 2011	5 199	5 137 (98.8)	62 (1.2)	133 (2.6)	11 (17.7)
Timor Leste 2009/10	9 472	9 308 (98.3)	164 (1.7)	162 (1.7)	20 (12.2)
**African Region (AFR)**	**302 508**	**292 396 (96.7)**	**10** **112 (3.3)**	**6469 (2.2)**	**1296 (12.8)**
Benin 2011/12	12 923	12 321 (95.3)	602 (4.7)	203 (1.6)	62 (10.3)
Burkina Faso 2010	14 026	13 546 (96.6)	480 (3.4)	248 (1.8)	71 (14.8)
Burundi 2010	7 405	7 237 (97.7)	168 (2.3)	157 (2.2)	19 (11.3)
Cameroon 2011	11 006	10 536 (95.7)	470 (4.3)	233 (2.2)	53 (11.3)
Chad 2004	5 071	4 961 (97.8)	110 (2.2)	129 (2.6)	16 (14.5)
Comoros 2012	3 082	2 956 (95.9)	126 (4.1)	49 (1.7)	13 (10.2)
Congo (Brazzaville 2011/12)	9 005	8 647 (96.0)	358 (4.0)	127 (1.5)	32 (8.9)
Congo (Democratic Republic 2013/14)	17 647	17 071 (96.7)	576 (3.3)	367 (2.1)	70 (12.1)
Cote d’Ivoire 2011/12	7 349	7 029 (95.7)	320 (4.3)	196 (2.8)	63 (19.7)
Ethiopia 2011	11 166	10 852 (97.2)	314 (2.8)	299 (2.8)	62 (19.8)
Gabon 2012	5 849	5 621 (96.1)	228 (3.9)	83 (1.5)	29 (12.8)
Gambia 2013	7 929	7 687 (96.9)	242 (3.1)	129 (1.7)	23 (9.5)
Ghana 2008	2 881	2 759 (95.8)	122 (4.2)	76 (2.7)	12 (9.9)
Guinea 2012	6 600	6 346 (96.2)	254 (3.8)	145 (2.3)	35 (13.8)
Kenya 2008/09	5 862	5 694 (97.1)	168 (2.9)	133 (2.3)	24 (14.2)
Lesotho 2009	3 739	3 641 (97.4)	98 (2.6)	114 (3.1)	20 (20.4)
Liberia 2013	7 224	6 968 (96.5)	256 (3.5)	148 (2.1)	29 (11.4)
Madagascar 2008/09	12 000	11 790 (98.3)	210 (1.7)	225 (1.9)	28 (13.3)
Malawi 2010	18 867	18 133 (96.1)	734 (3.9)	432 (2.4)	90 (12.2)
Mali 2012/13	9 873	9 567 (96.9)	306 (3.1)	261 (2.7)	35 (11.4)
Mozambique 2011	10 607	10 209 (96.3)	398 (3.7)	264 (2.6)	57 (14.3)
Niger 2012	11 820	11 482 (97.1)	338 (2.9)	192 (1.7)	33 (9.8)
Nigeria 2013	29 573	28 607 (96.7)	966 (3.3)	840 (2.9)	148 (15.3)
Rwanda 2010	8 678	8 432 (97.2)	246 (2.8)	163 (1.9)	34 (13.8)
Sao Tome and Principe 2008/2009	1 871	1 797 (96.0)	74 (4.0)	17 (1.0)	3 (4.0)
Senegal 2014	6 630	6 368 (96.1)	262 (3.9)	85 (1.3)	23 (8.8)
Sierra Leone 2013	10 997	10 597 (96.4)	400 (3.6)	333 (3.1)	52 (13.0)
Swaziland 2006/07	2 598	2 536 (97.6)	62 (2.4)	54 (2.1)	7 (11.3)
Tanzania 2010	7 705	7 495 (97.3)	210 (2.7)	153 (2.0)	33 (15.7)
Togo 2013/14	6 700	6 422 (95.8)	278 (4.2)	138 (2.1)	29 (10.4)
Uganda 2011	7 535	7 309 (97.0)	226 (3.0)	155 (2.1)	25 (11.1)
Zambia 2007	12 971	12 591 (97.1)	380 (2.9)	222 (1.8)	46 (12.1)
Zimbabwe 2010/11	5 319	5 189 (97.6)	130 (2.4)	99 (1.9)	20 (15.4)
**American Region (AMR)**	**58 922**	**57 956 (98.4)**	**966 (1.6)**	**729 (1.2)**	**83 (8.6)**
Bolivia 2008	8 353	8 255 (98.8)	98 (1.2)	146 (1.8)	13 (13.4)
Colombia 2010	17 589	17 337 (98.6)	252 (1.4)	139 (0.8)	16 (6.3)
Dominican Republic 2013	3 661	3 581 (97.8)	80 (2.2)	49 (1.4)	6 (7.5)
Guyana 2009	2 147	2 103 (98.0)	44 (2.0)	40 (1.9)	1 (2.3)
Haiti 2012	6 912	6 728 (97.3)	184 (2.7)	139 (2.1)	32 (17.4)
Honduras 2011/12	10 730	10 576 (98.6)	154 (1.4)	131 (1.2)	10 (6.5)
Peru 2012	9 530	9 376 (98.4)	154 (1.6)	85 (0.9)	5 (3.2)
**Eastern Mediterranean Region (EMR)**	**43 217**	**41 937 (97.0)**	**1280 (3.0)**	**756 (1.8)**	**109 (8.5)**
Egypt 2014	15 607	15 031 (96.3)	576 (3.7)	137 (0.9)	32 (5.6)
Jordan 2012	10 207	9 891 (96.9)	316 (3.1)	96 (1.0)	21 (6.6)
Morocco 2003/04	6 029	5 863 (97.3)	166 (2.7)	105 (1.8)	21 (12.6)
Pakistan 2012/13	11 374	11 152 (98.0)	222 (2.0)	418 (3.7)	35 (15.7)
**Western Pacific Region (WPR)**	**15 079**	**14 881 (98.7)**	**198 (1.3)**	**242 (1.6)**	**14 (7.1)**
Cambodia 2010	7 992	7 884 (98.7)	108 (1.3)	165 (2.1)	9 (8.4)
Philippines 2013	7 087	6 997 (98.7)	90 (1.3)	77 (1.1)	5 (5.5)
**Total**	**536 179**	**521 867 (97.3)**	**14 312 (2.7)**	**10 530 (2.0)**	**1 739 (12.1)**

This data set for 60 countries presented an early newborn mortality (number of deaths per 100 livebirths) of 2% in singletons and of 12.1% in twins. Based on available DHS data from selected countries, SEAR and AFR were the WHO regions with the highest mortality for both singletons (2.2 early neonatal deaths for 100 livebirths in both regions) and twins (14.6 and 12.8 deaths for 100 livebirths, respectively). On the other hand, AMR and EUR reported the lowest mortality for singletons (1.2 and 1.3 early neonatal deaths for 100 livebirths, respectively) while WPR reported the lowest mortality for twins (7.1 deaths for 100 livebirths) (**Table 2**).

There is a significant higher percentage of twin pregnancies in older mother (35 years or older) and those with higher parity (**Table 3**).

**Table 3:** Selected maternal characteristics of twin and singleton pregnancies in 60 low- and middle-income countries from Demographic and Health Survey (DHS) data

	Mothers of twins (N = 7 156)	Mothers of singletons (N = 521 867)	χ^2^ *P*-value
**Maternal age (years):**			
<18	36 (0.5%)	7828 (1.5%)	<0.001
18-34	5038 (70.4%)	406 013 (77.8%)	
35 or more	2082 (29.1%)	108 026 (20.7%)	
**Education:**			
No education	2624 (36.9%)	176 894 (33.9%)	<0.001
Primary	2236 (31.5%)	174 807 (33.5%)	
Secondary and more	2256 (31.6%)	170 110 (32.6%)	
*Missing*	*40*	*56*	
**Parity:**			
0	1195 (16.7%)	137 773 (26.4%)	<0.001
1-3	3492 (48.8%)	258 846 (49.6%)	
4 or more	2469 (34.4%)	125 248 (24.0%)	
**Number of ANC visits:**			
0	433 (7.6%)	36 051 (8.8%)	<0.001
1-3	1299 (22.8%)	85 210 (20.8%)	
4 or more	3964 (69.6%)	288 814 (70.5%)	
*Missing*	*1 460*	*111 792*	

**Table 4** shows crude and adjusted ORs for early neonatal mortality among twins when compared to singletons. The pooled adjusted OR (95% CI) was found to be 7.6 (7.0-8.3). All countries except Azerbaijan, Guyana, and Ukraine had statistically significant associations. In addition, Armenia and Moldova had no deaths among twins rendering it impossible to determine OR.

**Table 4:** Odds ratio (95% confidence intervals) of early neonatal deaths in twins vs singleton pregnancies: crude, adjusted for socio-economic and pregnancy covariates, and adjusted analysis for low birth weight (<2500 g) for countries with less than 20% missing data on weight at birth.

Regions, Countries, survey years	OR (95% CI) unadjusted	OR (95% CI) adjusted*	OR (95% CI) adjusted†
**All countries (n = 60)**	6.7 (6.4-7.1)	7.6 (7.0-8.3)	‡
**Countries with data on LBW (n = 20)**	7.5 (6.4-8.8)	7.4 (6.3-8.7)	2.8 (2.2-3.5)
**Pooled European Region (n = 8)**	7.9 (5.5-11.6)	8.9 (6.0-13.2)	‡
**Pooled European Region with data on LBW (n = 7)**	8.2 (5.6-11.9)	9.3 (6.2-13.8)	2.8 (1.6-4.9)
Albania 2008/09	11.7 (3.2-43.6)	10.8 (2.7-43.2)	3.0 (0.4-20.4)
Armenia 2010	‡	‡	‡
Azerbaijan 2006	3.0 (0.7-12.8)	1.6 (0.2-12.5)	‡
Kyrgyz Republic 2012	7.9 (3.6-17.0)	14.3 (6.2-33.1)	3.0 (1.2-7.6)
Moldova 2005	‡	‡	‡
Tajikistan 2012	12.7 (6.6-24.6)	16.3 (7.6-34.8)	4.5 (1.6-12.9)
Turkey 2003	8.5 (3.9-18.6)	12.9 (5.7-29.0)	§
Ukraine 2007	6.7 (0.8-56.6)	4.8 (0.5-47.9)	6.5 (0.4-21.6)
**Pooled South East Asian Region (n = 6)**	7.6 (6.5-8.8)	7.5 (6.3-8.8)	§
**Pooled South East Asian Region with data on lbw (n = 2)**	11.5 (8.0-16.4)	12.4 (8.3-18.7)	2.7 (1.5-4.9)
Bangladesh 2011	7.8 (4.9-12.3)	7.7 (4.7-12.5)	§
India 2005/06	6.4 (5.1-8.0)	7.8 (6.2-9.8)	§
Indonesia 2012	11.5 (8.0-16.4)	12.4 (8.3-18.7)	2.7 (1.5-4.9)
Maldives 2009	20.0 (8.3-48.2)	23.8 (8.1-69.7)	‡
Nepal 2011	8.3 (4.2-16.2)	8.4 (4.0-17.6)	§
Timor Leste 2009/10	7.8 (4.8-12.8)	7.6 (4.5-12.9)	§
**Pooled African Region (n = 33)**	6.6 (6.2-7.0)	6.6 (6.2-7.1)	§
**Pooled African Region with data on lbw (n = 4)**	7.1 (5.4-9.3)	7.4 (5.6-9.8)	3.6 (2.5-5.4)
Benin 2011/12	6.8 (5.1-9.2)	6.9 (5.0-9.6)	§
Burkina Faso 2010	9.3 (7.0-12.3)	11.5 (8.4-15.5)	§
Burundi 2010	5.7 (3.5-9.5)	6.9 (4.1-11.6)	§
Cameroon 2011	5.6 (4.1-7.7)	6.7 (4.7-9.4)	§
Chad 2004	6.4 (3.6-11.1)	7.1 (4.0-12.7)	§
Comoros 2012	6.7 (3.6-12.8)	5.8 (2.7-12.4)	§
Congo (Brazzaville 2011/12)	6.6 (4.4-9.8)	7.3 (4.8-11.1)	5.2 (3.1-9.0)
Congo (Democratic Republic 2013/14)	6.3 (4.8-8.2)	7.7 (5.8-10.3)	§
Cote d Ivoire 2011/12	8.5 (6.3-11.6)	9.0 (6.5-12.6)	§
Ethiopia 2011	8.7 (6.4-11.8)	8.0 (5.8-11.1)	§
Gabon 2012	9.8 (6.2-15.3)	12.7 (7.7-21.1)	3.0 (1.5-6.3)
Gambia 2013	6.1 (3.8-9.7)	7.5 (4.6-12.2)	§
Ghana 2008	3.9 (2.0-7.3)	4.0 (2.0-7.8)	§
Guinea 2012	6.8 (4.6-10.1)	7.6 (5.0-11.4)	§
Kenya 2008/09	6.9 (4.3-11.0)	6.7 (4.1-10.9)	§
Lesotho 2009	7.9 (4.7-13.4)	8.4 (4.9-14.6)	§
Liberia 2013	5.9 (3.9-9.0)	7.4 (4.8-11.4)	§
Madagascar 2008/09	7.9 (5.2-12.0)	9.5 (6.1-14.7)	§
Malawi 2010	5.7 (4.5-7.3)	6.8 (5.2-8.9)	§
Mali 2012/13	4.6 (3.2-6.7)	5.4 (3.6-7.9)	§
Mozambique 2011	6.3 (4.6-8.5)	8.6 (6.1-12.1)	§
Niger 2012	6.4 (4.3-9.4)	6.4 (4.3-9.6)	§
Nigeria 2013	6.0 (4.9-7.2)	6.8 (5.6-8.2)	§
Rwanda 2010	8.1 (5.5-12.1)	8.9 (5.8-13.5)	§
Sao Tome and Principe 2008/2009	4.4 (1.3-15.4)	10.7 (2.5-45.8)	1.9 (0.3-13.6)
Senegal 2014	7.1 (4.4-11.5)	8.1 (4.9-13.5)	§
Sierra Leone 2013	4.6 (3.4-6.3)	6.2 (4.4-8.5)	§
Swaziland 2006/07	5.8 (2.5-13.4)	7.5 (3.2-17.8)	1.8 (0.4-8.7)
Tanzania 2010	8.9 (6.0-13.4)	9.9 (6.4-15.1)	§
Togo 2013/14	5.3 (3.5-8.0)	5.6 (3.6-8.7)	§
Uganda 2011	5.8 (3.7-9.0)	5.8 (3.7-9.2)	§
Zambia 2007	7.7 (5.5-10.8)	10.0 (7.0-14.3)	§
Zimbabwe 2010/11	9.3 (5.6-15.7)	10.4 (6.0-18.1)	§
**Pooled American Region (n = 7)**	7.4 (5.8-9.3)	8.4 (6.5-10.9)	§
**Pooled American Region with data on lbw (n = 5)**	5.7 (4.0-8.3)	6.5 (4.5-9.4)	1.9 (1.2-3.3)
Bolivia 2008	8.6 (4.7-15.8)	10.8 (5.7-20.5)	3.0 (1.0-8.7)
Colombia 2010	8.3 (4.9-14.2)	12.2 (5.3-27.8)	§
Dominican Republic 2013	5.8 (2.4-14.1)	5.5 (2.2-13.7)	2.2 (0.8-6.4)
Guyana 2009	1.2 (0.2-9.1)	1.4 (0.2-10.6)	†
Haiti 2012	10.0 (6.6-15.1)	11.4 (7.3-17.9)	§
Honduras 2011/12	5.5 (2.8-10.7)	6.5 (3.3-12.8)	1.2 (0.3-4.1)
Peru 2012	3.7 (1.5-9.2)	4.0 (1.6-10.1)	1.0 (0.3-3.4)
**Pooled Eastern Mediterranean Region (n = 4)**	5.1 (4.1-6.2)	5.5 (4.5-6.9)	§
**Pooled Eastern Mediterranean Region with data on LBW (n = 1)**	7.3 (4.5-11.8)	7.9 (4.8-13.0)	2.1 (1.1-4.1)
Egypt 2014	6.4 (4.3-9.5)	8.2 (5.4-12.3)	§
Jordan 2012	7.3 (4.5-11.8)	7.9 (4.8-13.0)	2.1 (1.1-4.1)
Morocco 2003/04	7.9 (4.8-13.0)	7.4 (4.3-12.7)	§
Pakistan 2012/13	4.8 (3.3-6.9)	5.0 (3.4-7.5)	§
**Pooled Western Pacific Region (n = 2)**	4.6 (2.6-8.0)	4.8 (2.7-8.5)	§
**Pooled Western Pacific Region with data on LBW (n = 1)**	5.2 (2.1-13.2)	7.9 (3.0-20.9)	4.9 (1.1-22.2)
Cambodia 2010	4.2 (2.1-8.6)	4.3 (2.1-8.7)	§
Philippines 2013	5.2 (2.1-13.2)	7.9 (3.0-20.9)	4.9 (1.1-22.2)

OR – odds ratio, CI – confidence interval, LBW – low-birth weight

*Adjusted for mode of delivery, wealth, age, birth order, birth spacing, at least 1ANC, education and rural\urban residence.

†Adjusted for mode of delivery, wealth, age, birth order, birth spacing, ANC, education and rural\urban residence, and for low birthweight.

‡No deaths present for analysis.

§>20% missing data on birthweight.

In the subgroup analysis for the 20 countries with less than 20% missing data on birth weight, was associated with low birth weight significantly increased risk (aOR = 2.8; 95% CI = 2.2-3.5) of early newborn mortality of twins vs singletons. Low birth weight twins and singletons had 4.0 (2.0-8.0) and 7.6 (6.0-9.5) times the OR of early mortality, respectively, compared with twins and singletons of normal birth weight.

The overall proportion of twin births in health facilities was almost 70%. This proportion was highest in EUR (91.2%) and lowest in SEAR (56.8%) (**Table 5**). The average rate of caesarean section in twins was 17.5%; highest in AMR (55%) and EMR (51.7%) and lowest in WPR where only 11.6% of twin deliveries were through CS.

**Table 5:** Place of delivery and Caesarean section among the under-study twins born in in the five years preceding 60 low and middle-income Demographic and Health Survey (DHS) countries between 2005 and 2014

Country, survey years	Place of delivery			Mode of delivery	
	**Home-no SBA**	**Home-SBA**	**Health facility**	**Vaginal**	**Caesarean section**
**Total**	**3774 (27.0%)**	**494 (3.5%)**	**9828 (69.5%)**	**11** **548 (82.5%)**	**2452 (17.5%)**
**Pooled European Region**	**24 (6.6%)**	**8 (2.2%)**	**334(91.2%)**	**292 (79.1%)**	**74 (20.9%)**
Albania 2008/09	*	*	36 (100%)	26 (72.2%)	10 (27.8%)
Armenia 2010	*	*	20 (100%)	18 (90.0%)	2 (10.0%)
Azerbaijan 2006	4 (14.3%)	4 (14.3%)	20 (71.4%)	26 (86.2%)	2 (13.8%)
Kyrgyz Republic 2012	*	*	80 (100%)	64 (80.0%)	16 (20.0%)
Moldova 2005	*	*	18 (100.0%)	14 (77.8%)	4 (22.2%)
Tajikistan 2012	8 (9.4%)	2 (2.4%)	76 (88.2%)	82 (95.4%)	4 (4.6%)
Turkey 2003	12 (16.7%)	2 (2.8%)	58 (80.5%)	40 (54.2%)	32 (45.8%)
Ukraine 2007	*	*	26 (100%)	22 (84.6%)	4 (15.4%)
**Pooled South East Asian Region**	**462 (33.6%)**	**130 (9.6%)**	**778 (56.8%)**	**1080 (78.7%)**	**292 (21.3%)**
Bangladesh 2011	68 (46.2%)	6 (4.1%)	70 (49.7%)	100 (69.7%)	44 (30.3%)
India 2005/06	230 (33.0%)	52 (7.6%)	414 (59.4%)	554 (79.6%)	142 (20.4%)
Indonesia 2012	48 (18.8%)	46 (18.0%)	160 (63.2%)	194 (76.1%)	60 (23.9%)
Maldives 2009	*	*	52 (100.0%)	24 (50.9%)	28 (49.1%)
Nepal 2011	24 (40.7%)	2 (3.4%)	34 (55.9%)	56 (93.4%)	4 (6.6%)
Timor Leste 2009/10	94 (56.8%)	24 (14.8%)	46 (28.4%)	150 (91.5%)	14 (8.5%)
**Pooled African Region**	**2972 (29.8%)**	**304 (3.1%)**	**6686 (67.1%)**	**8954 (91.1%)**	**920 (8.9%)**
Benin 2011/12	38 (6.2%)	6 (1.2%)	552 (92.6%)	542 (92.2%)	48 (7.8%)
Burkina Faso 2010	114 (24.0%)	4 (0.6%)	362 (75.4%)	436 (93.7%)	32 (6.2%)
Burundi 2010	44 (26.5%)	*	120 (73.5%)	142 (88.7%)	22 (11.3%)
Cameroon 2011	98 (21.3%)	20 (4.6%)	340 (74.1%)	426 (94.9%)	26 (5.1%)
Chad 2004	70 (65.4%)	14 (13.1%)	24 (21.5%)	104 (99.1%)	4 (0.9%)
Comoros 2012	18 (13.8%)	8 (6.9%)	94 (79.3%)	106 (89.0%)	14 (11.0%)
Congo (Brazzaville 2011/12)	32 (9.3%)	6 (1.4%)	320 (89.3%)	330 (92.2%)	28 (7.8%)
Congo (Democratic Republic 2013/14)	116 (20.2%)	20 (3.5%)	440 (76.3%)	510 (90.6%)	56 (9.4%)
Cote d Ivoire 2011/12	128 (40.3%)	8 (2.5%)	184 (57.2%)	300 (94.7%)	20 (5.3%)
Ethiopia 2011	258 (84.0%)	2 (0.6%)	50 (15.4%)	296 (96.2%)	14 (3.8%)
Gabon 2012	28 (12.6%)	2 (0.9%)	194 (86.5%)	182 (80.4%)	42 (19.6%)
Gambia 2013	52 (21.7%)	4 (2.1%)	186 (76.2%)	226 (93.8%)	16 (6.2%)
Ghana 2008	12 (9.9%)	20 (16.5%)	90 (73.6%)	100 (82.6%)	22 (17.4%)
Guinea 2012	116 (46.5%)	32 (13.0%)	104 (40.5%)	238 (94.5%)	14 (5.5%)
Kenya 2008/09	80 (47.6%)	2 (0.6%)	86 (51.8%)	142 (84.6%)	26 (15.4%)
Lesotho 2009	32 (33.7%)	18 (17.3%)	48 (49.0%)	84 (86.7%)	14 (13.3%)
Liberia 2013	92 (36.1%)	8 (3.5%)	154 (60.4%)	220 (87.6%)	34 (12.4%)
Madagascar 2008/09	78 (36.7%)	28 (13.3%)	104 (50.0%)	200 (95.2%)	10 (4.8%)
Malawi 2010	176 (24.5%)	*	540 (75.5%)	658 (92.2%)	58 (7.8%)
Mali 2012/13	100 (33.1%)	10 (3.3%)	192 (63.6%)	282 (94.5%)	20 (5.5%)
Mozambique 2011	118 (31.4%)	*	256 (68.5%)	334 (89.9%)	40 (10.1%)
Niger 2012	154 (46.7%)	4 (0.9%)	176 (52.4%)	306 (92.8%)	28 (7.2%)
Nigeria 2013	404 (42.5%)	56 (5.9%)	496 (51.6%)	892 (93.3%)	64 (6.7%)
Rwanda 2010	56 (22.8%)	*	186 (77.2%)	192 (79.3%)	50 (20.7%)
Sao Tome and Principe 2008/2009	16 (21.6%)	*	58 (78.4%)	68 (91.9%)	6 (8.1%)
Senegal 2014	58 (22.1%)	2 (0.8%)	202 (77.1%)	230 (90.5%)	32 (9.5%)
Sierra Leone 2013	132 (33.5%)	16 (4.1%)	246 (62.4%)	366 (89.0%)	28 (11.0%)
Swaziland 2006/07	14 (22.9%)	*	48 (77.1%)	54 (87.1%)	8 (12.9%)
Tanzania 2010	82 (39.4%)	*	126 (60.6%)	184 (87.9%)	24 (12.1%)
Togo 2013/14	74 (26.5%)	*	204 (73.5%)	240 (86.0%)	38 (14.0%)
Uganda 2011	60 (26.7%)	4 (2.2%)	160 (71.1%)	198 (88.4%)	26 (11.6%)
Zambia 2007	88 (23.5%)	2 (0.5%)	286 (76.0%)	334 (88.6%)	42 (11.4%)
Zimbabwe 2010/11	34 (26.6%)	4 (2.3%)	92 (71.1%)	116 (90.0%)	14 (10.0%)
**Pooled American Region**	**140 (15.1%)**	**20 (2.2%)**	**764 (82.7%)**	**436 (44.9%)**	**488 (55.1%)**
Bolivia 2008	20 (20.6%)	8 (8.2%)	68 (71.1%)	52 (54.6%)	44 (45.4%)
Colombia 2010	6 (4.6%)	*	210 (97.7%)	58 (22.9%)	158 (77.1%)
Dominican Republic 2013	2 (2.5%)	*	78 (97.5%)	24 (28.7%)	56 (71.2%)
Guyana 2009	2 (4.6%)	*	42 (95.4%)	24 (53.5%)	20 (46.5%)
Haiti 2012	92 (51.1%)	*	90 (48.9%)	162 (87.5%)	20 (12.5%)
Honduras 2011/12	4 (2.6%)	12 (8.5%)	138 (88.9)	74 (47.4%)	80 (52.6%)
Peru 2012	14 (9.2%)	*	138 (90.8%)	42 (27.3%)	110 (72.7%)
**Pooled Eastern Mediterranean Region**	**128 (10.0%)**	**18 (1.4%)**	**1132 (88.6%)**	**612 (48.3%)**	**656 (51.7%)**
Egypt 2014	22 (4.0%)	6 (1.1%)	546 (94.9%)	180 (31.4%)	394 (68.6%)
Jordan 2012	4 (1.3%)	*	312 (98.7%)	116 (37.8%)	190 (62.2%)
Morocco 2003/04	40 (23.5%)	2 (1.2%)	124 (75.3%)	148 (89.7%)	18 (10.3%)
Pakistan 2012/13	62 (28.1%)	10 (4.5%)	150 (67.4%)	168 (75.8%)	54 (24.2%)
**Pooled Western Pacific Region**	**48 (24.5%)**	**14 (7.1%)**	**134 (68.4%)**	**174 (88.4%)**	**22 (11.6%)**
Cambodia 2010	30 (27.1%)	10 (10.3%)	68 (62.6%)	96 (89.7%)	12 (10.3%)
Philippines 2013	18 (21.3%)	4 (4.5%)	66 (74.2%)	78 (86.8%)	10 (13.2%)

SBA – skilled birth attendant

*Missing data.

Overall, sixteen countries had high rates of cesarean sections (>15%), 36 middle (5%-15%) and eight low (<5%). For twins 22 countries had high rates of cesarean sections (defined for this analysis as those >15%), 34 had middle rates (5%-15%) and four had low rates (<5%), (**Table 5**).

After adjusting for maternal age, maternal education, household wealth, rural\urban residence, sex, birth spacing and desired pregnancy, twins delivered vaginally in health facility had a statistically significantly increased risk for early newborn mortality (pooled aOR = 2.1; CI = 1.5-3.1) compared to twins delivered by caesarean section (**Table 6**). This statistically significant association became more pronounced (aOR = 4.8; 95% CI = 2.4-9.4) for countries with high rates of CS deliveries but lost significance for middle and low-rates countries (**Table 6**).

**Table 6:** Pooled and stratified crude and adjusted odds ratios (95% confidence Intervals) for early neonatal deaths in vaginal vs caesarean section deliveries in 318 823 singleton and in 9828 twin health facility births from 60 countries

Rates of Caesarean Sections	Type of pregnancy	OR (95% CI) unadjusted	OR (95% CI) adjusted*
Pooled	Singleton	0.8 (0.7-0.9)	0.7 (0.6-0.8)
	Twin	2.2 (1.8-2.7)	2.1 (1.5-3.1)
Low (<5%)	Singleton	0.6 (0.5-0.7)	0.5 (0.4-0.6)
	Twin	1.0 (0.8-1.3)	1.1 (0.7-1.8)
Medium (5%-15%)	Singleton	0.7 (0.6-0.9)	0.5 (0.4-0.6)
	Twin	2.1 (0.9-4.5)	2.1 (0.9-4.6)
High (>15%)	Singleton	0.7 (0.5-0.8)	0.6 (0.5-0.8)
	Twin	4.8 (2.4-9.6)	4.8 (2.4-9.4)

OR – odds ratio, CI – confidence interval

*Adjusted for wealth, age, sex, birth spacing, wanted pregnancy, education and rural\urban residence.

The same adjusted logistic regression performed among singleton deliveries showed a pooled (aOR = 0.7; CI = 0.6-0.8) protective effect of vaginal birth in health facility compared with a caesarean section; this beneficial effect held true for across the three rates-categories (**Table 6**).

In twin pregnancies, home delivery without SBA was associated with increased mortality both compared with delivering at home with SBA (aOR = 1.3; CI = 1.0-1.8) and with vaginal birth in health facility (aOR = 1.7; CI = 1.4-2.0). Home deliveries with SBA were at increased risk of early mortality compared with vaginal birth in health facility (aOR = 1.5; CI = 1.0-2.3) (**Table 7**).

**Table 7:** Crude and adjusted odds ratios (95% confidence Intervals) for early neonatal deaths in 11 651 twin births: (a) home without skilled birth attendance vs vaginal health facility deliveries; (b) home with skilled birth attendance vs vaginal health facility deliveries; (c) home without vs home with skilled birth attendance deliveries

	OR (95% CI) unadjusted	OR (95% CI) adjusted*
Non-SBA home delivery vs health facility vaginal delivery	1.7 (1.5-1.9)	1.7 (1.4-2.0)
SBA home delivery vs health facility vaginal delivery	1.2 (1.0-1.6)	1.5 (1.0-2.3)
Non-SBA home delivery vs SBA home delivery	1.4 (1.1-1.8)	1.3 (1.0-1.8)

OR – odds ratio, CI – confidence interval, SBA – skilled birth attendant

*Adjusted for wealth, age, sex, birth spacing, wanted pregnancy, education and rural\urban residence.

## DISCUSSION

Individual level data analysis from 60 low- and middle-income countries using population-based nationally-representative surveys revealed twins had around 3 times increased odds of death compared to singletons after adjusting for birth weight. CS was associated with a significant protective effect in twins (OR = 2.1; 95% CI = 1.5-3.1), however, when stratifying by national CS rates, the significance remained only in the countries where the rate of CS was 15% or higher. In contrast, delivery by CS was associated with increased risk of mortality among singletons regardless of section rates.

These associations need cautious interpretation. The DHS surveys do not provide information about factors that may lead to a CS (eg, obstructed labour, fetal distress or pre-eclampsia/eclampsia) and thus we could not control for other morbidity associated beyond the increased inherent risk of a multiple pregnancy. Determining whether the CS was the cause or the consequence of the morbidity or mortality suffered by the mother or the newborn is a chronic challenge in this type of analysis.

This study has several limitations. No information was available regarding medical conditions (chronic hypertension, malaria), obstetric antenatal conditions (prelabour rupture of membranes, pregnancy-induced hypertension, pre-eclampsia, eclampsia, vaginal bleeding in the second half of pregnancy) and fetal presentation which have an impact in the mode of delivery and the outcome of pregnancy. Similarly, no data on race and smoking status were available as well as lack of information on the use of antenatal ultrasound with early detection of twins and its impact on the outcome.

No information was available on who the first and who was the second twin was, which would have an impact on the interpretation regarding potential mortality in retained second twin. One additional limitation resulted from reporting bias and the lack of information on women who died during delivery, likely associated with increased risk of early neonatal deaths. However, given the large number of neonatal deaths per every maternal death, the overall impact maternal deaths is small.

We lack data on chorionicity which is it well known to have impact on neonatal outcomes [22]. We were not able either to measure the influence of twin birth interval [23,24] nor the influence of difference in weight between first and second twins [25].

Lastly, in consideration of the cross-sectional nature of DHS, there is the possibility of underreporting and recall bias. The chance of underreporting of live births, recognized problem even in industrializes countries [26], would be higher for one of twins than for singleton, which translates in an underestimation of the excess mortality of twins [27].

Literature has provided discordant conclusions so far on the safest mode of delivery for twins: several studies generated significant protective results on perinatal outcomes with CS in case of elective CS vs vaginal delivery or emergency CS [28,29]. However, a recent systematic review [30] of two randomized trials found no clear evidence of benefits from planned CS for term twin pregnancies with leading cephalic presentation.

Most previous studies were conducted in high-income countries where access to quality care during CS is almost universal. However, our results lead to the same conclusions of the few studies conducted in low- and middle-income countries [30], which supports the need for timely access to safe caesarean births for twin pregnancies [31].

Despite the significant overall beneficial effect on early neonatal mortality when twins are delivered by CS in this data set, when stratifying countries according to CS rates in twins, this effect was maintained only in countries with rates of CS in twins of more than 15%. This may be explained by an important reduction of adverse outcomes for retained second twins, which can occur in up to one fourth twin deliveries [32], as well as fewer deaths in case of non-vertex first twin presentation [33]; it could also reflect a more widespread availability of health facilities performing CS for women experiencing obstetrical complications such as obstructed labor or eclampsia, which would reduce fetal distress.

In countries with lower CS rates (<15%), the lack of benefit may be due to a limited timely-access to emergency CS at population level. This may reflect not only the insufficient number of facilities performing sections but also the lack of skills among the health care providers, poor infrastructure and equipment, as well as routines such as early discharge from health facility after birth. The fewer CS conducted could be those in desperate situations, dangerous conditions without appropriate means rendering almost impossible to achieve a good outcome and thus limiting the impact of CS on early mortality.

That singleton deliveries by CS had an associated higher risk of mortality is discordant with findings from all-deliveries ecological studies [34-37], which show that newborn mortality decrease as CS increases up to 10%-15% [38], in line with the World Health Organization recommendations [39-40].

However, we should highlight the methodological bias [41] of the above-mentioned studies since no individual level data was used and only ecological associations were measured.

A similar study [15] using data from 46 Demographic and Health Surveys to assess the association between CS and newborn mortality showed significant increased OR of neonatal mortality among singletons delivered by CS in countries with low or middle CS rates. The same study speculated on the possible negative role of poor infrastructure in health facilities, lack of surgical and poor neonatal care underscoring the need to avoid unnecessary CS especially in settings that lack the facilities and/or capacity to properly conduct safe surgery and treat surgical complications, as recommended by the 2015 WHO statement [39-40].

Home delivery of twins with or without SBA was associated with increased risk of early neonatal mortality compared with delivering in a health facility through vaginal delivery aOR = 1.5 (95% CI = 1.0-2.3) and aOR = 1.7 (1.4-2.0), respectively. Home births without an SBA were associated with increased early neonatal mortality (OR 1.3; 95% CI = 1.0-1.8) compared with home births attended by SBA. This beneficial effect of institutional delivery on twins regardless of the availability of a SBA at home to give birth is not surprising for a wide range of factors which makes twins pregnancies more prone to adverse outcome, thus requiring higher capacity to manage complications at birth. The lesser benefit of SBA in home births when compared to non-SBA births is not surprising.

Twin pregnancies are associated with 7.6 (95% CI = 7.0-8.3) increased early newborn mortality compared to singleton pregnancies. This association was found in 55 out of 60 countries-analyzed. After adjusting for birthweight, the association decreased to 2.8 (95% CI = 2.2-3.5). Our findings on increased overall early neonatal mortality in twins compared to singleton births is in line with previous findings [42,43] and it is due to increased rates obstetric and perinatal complications [7-9,44] and several innate characteristics such as premature separation of placenta [45]. Other possible reasons include feeding and other cultural practices, especially in Africa and in Asia [46,47], whereby one of the twin babies could get preferential care. Concerning the differential mortality due to low birthweight between twins and singleton births we may infer a confounding role played by gestational age: twins are LBW because of constricted growth and at any given stage their organs are more mature than similarly LBW singletons likely because at higher gestational age.

## CONCLUSIONS

Our findings suggest the need to identify twin pregnancies prior to labor to centralize care at birth; there is also a need to improve the access to safe CS for twin pregnancies in low- and middle-income countries where skills and capacity for the care and management of twin pregnancies as well as the potential complications may not exist. This is particular important for women in rural areas of countries of sub-Saharan Africa due to the enormous gap in the availability of skilled workers able to perform surgery [48]. In the absence of access to safe CS and/or capacity to treat and manage its complications or those of inherent to twin pregnancies, risks and benefits must be carefully balanced due to the negative potential consequences of unsafe surgery.

These medical aspects should be explored together with cultural beliefs, ie, related to birth order and gender, which may play an important role in the twins’ survival.
